# Stress-Induced Reorganization of the Mycobacterial Membrane Domain

**DOI:** 10.1128/mBio.01823-17

**Published:** 2018-01-23

**Authors:** Jennifer M. Hayashi, Kirill Richardson, Emily S. Melzer, Steven J. Sandler, Bree B. Aldridge, M. Sloan Siegrist, Yasu S. Morita

**Affiliations:** a Department of Microbiology, University of Massachusetts, Amherst, Massachusetts, USA; b Department of Microbiology and Molecular Biology, Tufts University School of Medicine, Boston, Massachusetts, USA; c Department of Biomedical Engineering, Tufts University, Medford, Massachusetts, USA; Washington University in St. Louis School of Medicine

**Keywords:** Mycobacterium, cell envelope, membrane proteins, membranes, peptidoglycan, stress response

## Abstract

Cell elongation occurs primarily at the mycobacterial cell poles, but the molecular mechanisms governing this spatial regulation remain elusive. We recently reported the presence of an intracellular membrane domain (IMD) that was spatially segregated from the conventional plasma membrane in *Mycobacterium smegmatis*. The IMD is enriched in the polar region of actively elongating cells and houses many essential enzymes involved in envelope biosynthesis, suggesting its role in spatially restricted elongation at the cell poles. Here, we examined reorganization of the IMD when the cells are no longer elongating. To monitor the IMD, we used a previously established reporter strain expressing fluorescent IMD markers and grew it to the stationary growth phase or exposed the cells to nutrient starvation. In both cases, the IMD was delocalized from the cell pole and distributed along the sidewall. Importantly, the IMD could still be isolated biochemically by density gradient fractionation, indicating its maintenance as a membrane domain. Chemical and genetic inhibition of peptidoglycan biosynthesis led to the delocalization of the IMD, suggesting the suppression of peptidoglycan biosynthesis as a trigger of spatial IMD rearrangement. Starved cells with a delocalized IMD can resume growth upon nutrient repletion, and polar enrichment of the IMD coincides with the initiation of cell elongation. These data reveal that the IMD is a membrane domain with the unprecedented capability of subcellular repositioning in response to the physiological conditions of the mycobacterial cell.

## IMPORTANCE

Mycobacteria include medically important species, such as the human tuberculosis pathogen *Mycobacterium tuberculosis*. The highly impermeable cell envelope is a hallmark of these microbes, and its biosynthesis is a proven chemotherapeutic target. Despite the accumulating knowledge regarding the biosynthesis of individual envelope components, the regulatory mechanisms behind the coordinated synthesis of the complex cell envelope remain elusive. We previously reported the presence of a metabolically active membrane domain enriched in the elongating poles of actively growing mycobacteria. However, the spatiotemporal dynamics of the membrane domain in response to stress have not been examined. Here, we show that the membrane domain is spatially reorganized when growth is inhibited in the stationary growth phase, under nutrient starvation, or in response to perturbation of peptidoglycan biosynthesis. Our results suggest that mycobacteria have a mechanism to spatiotemporally coordinate the membrane domain in response to metabolic needs under different growth conditions.

## INTRODUCTION

*Mycobacterium tuberculosis* latently infects one-third of the world’s population, with 1.8 million deaths reported in 2015 alone, including 0.4 million deaths among HIV patients (1). A notable global health concern is the spread of multidrug-resistant strains, which account for about 4% of new cases and 21% of previously treated cases, highlighting a need to develop new chemotherapeutic strategies. An important control point of *M. tuberculosis* pathogenesis is the metabolic activities associated with cell growth, as evidenced by the fact that current first-line drugs target enzymatic reactions critical for this process. However, *M. tuberculosis* is not always actively growing during infection. In fact, when *M. tuberculosis* enters into nongrowing states in the host, it is subjected to an array of stresses, from starvation to changes in pH to oxidative stress by reactive oxygen species, all of which have been suggested to be key triggers of the growth state transition (2). How *M. tuberculosis* responds to these stresses, rearranges its cellular architecture and modified its metabolism, and persists in the host for decades remains an important question for the understanding of tuberculosis disease.

The thick and waxy cell envelope of mycobacteria is a unique feature that is critical for the successful establishment of infection. This multilayered structure is composed of an inner (plasma) membrane, a cell wall with peptidoglycan (PG) and arabinogalactan, and an outer membrane made of highly hydrophobic mycolic acids and glycolipids. The cell surface architecture is thought to provide the main permeability barrier against host immune attack and antibiotic penetration. In fact, the mycobacterial cell envelope is 3 orders of magnitude less permeable to β-lactam antibiotics than that of *Escherichia coli* (3). Furthermore, the structural defects in cell envelope components, such as lipomannan/lipoarabinomannan, phthiocerol dimycocerosate, and other mycolate-containing molecules, compromise the permeability barrier (4–7), highlighting the importance of understanding how mycobacteria create and maintain this complex surface structure.

In mycobacteria, including *M. tuberculosis* and the nonpathogenic model organism *Mycobacterium smegmatis*, cell elongation is thought to take place only at the poles of the cell. Such polar elongation was first indicated via spatially restricted labeling of BODIPY-vancomycin, a probe that binds to nascent PG, at the cell poles (8–10). Later, polar extension of the new cell envelope was directly demonstrated by monitoring dilution of old cell envelopes prelabeled with an amine-reactive Alexa Fluor 488 fluorescent dye (11). Furthermore, several cell envelope biosynthetic enzymes were found to be enriched in the subpolar zone of elongating mycobacterial cells (12). Recently, we demonstrated the presence of a growth pole-associated intracellular membrane domain (IMD) (13), which was initially described as the PMf (*p*lasma *m*embrane *f*ree of cell wall components) (14). The IMD can be purified by sucrose density gradient sedimentation, and the name PMf reflects the biochemical nature of this membrane domain, which contains purely membrane lipids without cell wall (CW) components, in contrast to the conventional PM. The conventional PM is tightly associated with the CW and can be separated from the IMD by a sucrose gradient; thus, the conventional PM is designated the PM-CW. The IMD is bound by many enzymes of cell envelope precursor biosynthesis, including phosphatidic acid, lipid II, galactan, and phosphatidylinositol mannoside, which mediate key steps to produce phospholipids, peptidoglycan, arabinogalactan, and lipo(arabino)mannan, respectively. Using fluorescent IMD-associated protein reporters (hemagglutinin [HA]-mCherry-GlfT2 and Ppm1-mNeonGreen-cMyc), we identified the IMD at the nascent growth poles of actively growing *M. smegmatis* cells. Our data support the role of the IMD in active elongation of the cell envelope at the cell poles, but it remains unknown how the IMD responds to nongrowing conditions.

In this study, we determined that the IMD is maintained as a membrane domain but is spatially reorganized in nongrowing cells. Using the above-mentioned IMD reporter strain, we tracked the *in vivo* behavior of the IMD by using fluorescence microscopy during the transition from growing to nongrowing conditions. First, we examined the natural transition of the IMD from logarithmic growth to stationary phase. Second, we tested the effects of nutrient deprivation in a starvation model. Third, we inhibited PG biosynthesis by chemical and genetic approaches to determine if the IMD rearrangement is triggered by reduced PG synthesis. Our microscopy data, combined with biochemical fractionation analysis results, suggest that the polar enrichment of the IMD is diminished in the absence of cellular growth, yet the membrane domain remains isolatable by sucrose density gradient fractionation. Furthermore, the polar enrichment of the IMD is regained when cells resume growth. These results implicate the IMD as a spatiotemporally dynamic subcellular structure that is critical, not only during active growth but also during the transition to quiescent stages.

## RESULTS

### Polar enrichment of the IMD is diminished during the stationary phase.

**FIG 1:**
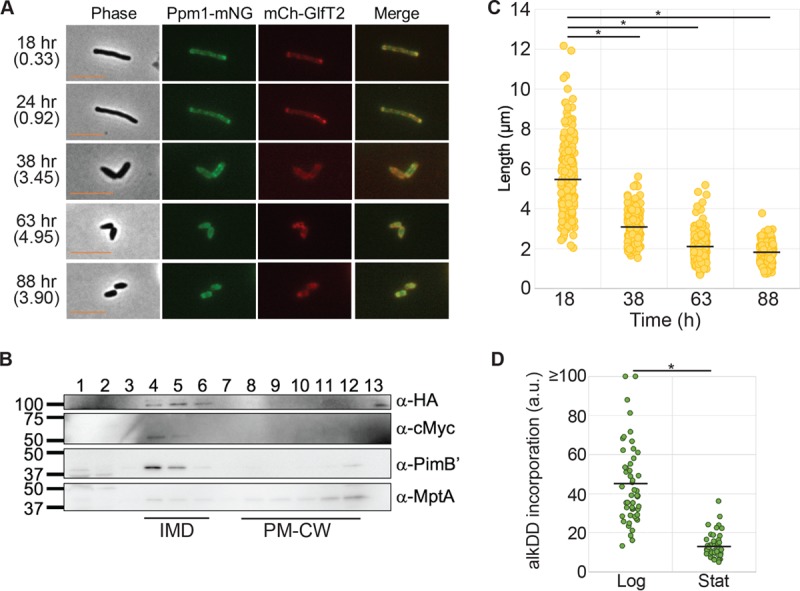
The IMD redistributes during stationary phase. (A) Fluorescence live imaging of the dual IMD marker strain expressing HA-mCherry-GlfT2 (mCh-GlfT2) and Ppm1-mNeonGreen-cMyc (Ppm1-mNG). Cells show reorganization of the IMD during stationary phase. Cell density (the OD_600_) is shown in parentheses to indicate the growth stage. Scale bar, 5 µm. (B) Western blotting detection of IMD markers after sucrose gradient sedimentation of stationary-phase cell lysate (48 h), demonstrating the biochemical isolation of the IMD. IMD markers were HA-mCherry-GlfT2 (anti-HA, 100 kDa), Ppm1-mNeonGreen-cMyc (anti-c-Myc, 59 kDa), and PimB′ (anti-PimB′, 42 kDa). The PM-CW marker was MptA (anti-MptA, 54 kDa). Note that the apparent molecular mass of MptA based on the SDS-PAGE was ~43 kDa (28). (C) Lengths of cells from logarithmic-phase growth (18 h) to stationary-phase growth (38, 63, and 88 h), showing the production of short cells. The black lines indicate averages of 202 cells. (D) Quantification of alkDADA incorporation normalized to cell sizes of stationary-phase cells (OD > 3.0), showing decreased *de novo* PG synthesis. The black lines indicate averages of 50 cells. *, *P* < 0.001.

In our previous study, we showed that the IMD was no longer detectable by sucrose density gradient in late stationary phase (150 h) (14), but little is known about its behavior during the growth phase transition. To begin dissecting the initial response of the IMD to growth-inhibiting conditions, we monitored the localization of this domain in the early stationary growth phase by using the previously established IMD reporter strain described above. In this strain, endogenous copies of the essential IMD-associated proteins galactosyltransferase GlfT2 and mannosyltransferase Ppm1 have been replaced with the fluorescent fusion proteins HA-mCherry-GlfT2 and Ppm1-mNeonGreen-cMyc, respectively (13). As previously observed, at the 18-h time point in the growth curve, when the cells are in logarithmic growth, the IMD markers were enriched at the growth poles. However, when the cells entered into stationary phase (38 h; optical density at 600 nm [OD_600_] of 3.45), the intense polar fluorescence of the IMD was diminished (Fig. 1A). This intensity change in IMD-associated proteins implied either that (i) the IMD was disassembled and proteins became randomly associated with the conventional plasma membrane (i.e., PM-CW) or formed aggregates within the cytoplasm or (ii) the IMD was spatially reorganized in the absence of polar growth. To distinguish between these two possibilities, we fractionated the cell lysate from a stationary-phase culture (48-h time point) along a sucrose density gradient (Fig. 1B). We found PimB′, an IMD marker, predominantly in the IMD fractions (4 to 6), with a small amount of this peripheral membrane protein released to the cytosol (fraction 1) as well as localized to the PM-CW (with MptA as a marker; fractions 8 to 12). Importantly, in addition to PimB′, both HA-mCherry-GlfT2 and Ppm1-mNeonGreen-cMyc continued to be detectable in the IMD fractions, despite the lack of active elongation of the cells. This implies that the IMD is maintained but spatially rearranged during the log-phase–stationary-phase transition.

In stationary phase, mycobacteria undergo cell division without cell elongation (15), creating a population of cells shorter in cell length (Fig. 1C). During this stationary-phase cell division, cells no longer elongate by adding new cell envelope components at their cell poles but continue to produce cell envelope components for septum synthesis. PG is a key component of the mycobacterial cell envelope and is essential for elongation at the cell poles as well as septum synthesis. A key step in PG synthesis is mediated by MurG, a glycosyltransferase that synthesizes lipid II from its precursor, lipid I. MurG is one of the proteins associated with the IMD proteome (13) and has been found enriched in the subpolar region of *M. smegmatis* cells (12). Therefore, we speculated that the spatial rearrangement of the IMD correlates with the location of PG synthesis. To address this question, we visualized *de novo* PG synthesis by using metabolic labeling. We incubated another reporter *M. smegmatis* strain expressing HA-mCherry-GlfT2 with alkyne-functionalized d-Ala-d-Ala dipeptide (alkDADA) (16) for 15 min and detected the probe by using copper-catalyzed azide-alkyne cycloaddition of a fluorescent azide label. In correlation with active cell envelope elongation, log-phase cells incorporated quantitatively more alkDADA into their cell wall than did stationary-phase cells (Fig. 1D). Furthermore, alkDADA was predominantly incorporated into cell poles, slightly distal to the apparent subpolar location of the IMD marker (see Fig. S1 in the supplemental material). In contrast, the fluorescent signal of *de novo* PG synthesis was decreased in stationary phase (Fig. 1D) and was localized more prominently in the septa (Fig. S1). Thus, the decrease in polar enrichment of the IMD during stationary phase correlated with the spatially rearranged nonpolar PG biosynthesis.

10.1128/mBio.01823-17.1 FIG S1  Reduced levels of PG synthesis continue at the septa when cells are no longer elongating and the IMD is delocalized from the pole. Logarithmically growing cells, with subpolar enrichment of IMD fluorescence (HA-mCherry-GlfT2), incorporate alkDADA at the cell poles. During stationary phase (54 h) and starvation (30 h), the IMD delocalizes from the pole and PG synthesis becomes more restricted to septum synthesis. Arrowhead, an example of occasional polar alkDADA fluorescence in stationary-phase cells. Scale bar, 5 µm. Download FIG S1, TIF file, 6.4 MB. Copyright © 2018 Hayashi et al. 2018 Hayashi et al. This content is distributed under the terms of the Creative Commons Attribution 4.0 International license.

### Starvation induces reorganization of the IMD to the sidewalls.

**FIG 2:**
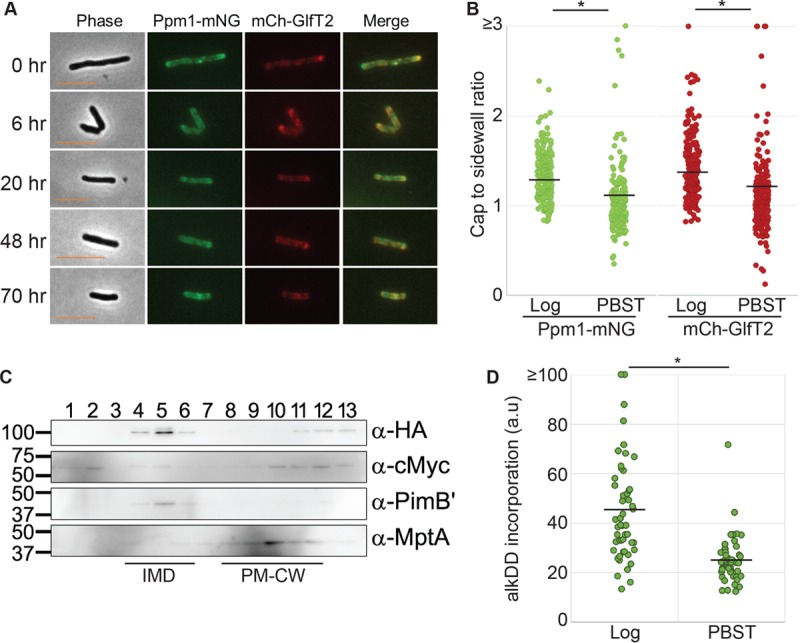
The IMD reorganizes along the sidewall of the cell body during PBST starvation. (A) Fluorescence live imaging of the dual IMD marker strain expressing HA-mCherry-GlfT2 (mCh-GlfT2) and Ppm1-mNeonGreen-cMyc (Ppm1-mNG). Exposure of actively growing cells to PBST led to the redistribution of the IMD. Scale bar, 5 µm. (B) Differences in polar fluorescence enrichment between actively growing (log) and 48-h-starved (PBST) cells. Polar enrichment was calculated as the ratio of mean fluorescence intensities between the polar cap and the sidewall region. The black lines indicate the average of 201 cells. (C) Western blotting detection of IMD proteins from sucrose gradient sedimentation demonstrated the biochemical isolation of the IMD in cells starved in PBST for 30 h. IMD markers were HA-mCherry-GlfT2 (anti-HA, 100 kDa), Ppm1-mNeonGreen-cMyc (anti-c-Myc, 59 kDa), and PimB′ (anti-PimB′, 42 kDa). The PM-CW marker was MptA (anti-MptA, 54 kDa). (D) Quantification of alkDADA incorporation, normalized to cell sizes of cells after 30 h of PBST starvation, showing decreased *de novo* PG synthesis. The black lines indicate the averages of 50 cells. *, *P* < 0.001.

Nutrient deprivation is a well-established model to study the stress response of mycobacteria, with early work dating back to the 1930s (17, 18). It is also relevant to *M. tuberculosis* pathogenesis, as tissue infected with *M. tuberculosis* in mice or human patients expresses genes involved in adaptation to nutrient limitation (19). Interestingly, *M. smegmatis* exposed to mild nutrient starvation in phosphate-buffered saline with Tween 80 (PBST) continues to divide without cell separation, resulting in a filamentous morphology with multiple nucleoids and multiple septa (20). This response to PBST suggests that septum biosynthesis continues even under limited nutrient availability conditions. These observations led us to question how the IMD responds to nutrient deprivation in *M. smegmatis*. We grew cells expressing HA-mCherry-GlfT2 and Ppm1-mNeonGreen-cMyc to logarithmic phase in normal Middlebrook 7H9 medium and then replaced the medium with PBST. As expected, the cells no longer grew upon nutrient depletion (Fig. S2A). Consistent with the previous report that PBST-treated cells do not undergo cell separation (20), the average cell length was maintained relatively comparable to that of actively growing cells over 70 h of starvation (Fig. 2A; Fig. S2B). After 20 h of starvation, the IMD foci were no longer enriched at the poles but were reorganized along the cell sidewall (Fig. 2A). We took the 48-h time point and quantified the polar IMD enrichment as the ratio of mean fluorescence intensities between the polar cap and sidewall region. As shown in Fig. 2B and Fig. S3, the polar enrichment of the IMD was significantly reduced over time during the PBST starvation, and this delocalization was already evident at the earliest (6 h) time point (Fig. S3). The IMD remains biochemically isolatable in a sucrose gradient even after 36 h under starvation conditions (Fig. 2C), as evidenced by the enrichment of HA-mCherry-GlfT2 and endogenous PimB′ in fractions 4 to 6. We note that there was a minor and more significant relocation of HA-mCherry-GlfT2 and Ppm1-mNeonGreen-cMyc to the denser part of the sucrose gradient, suggesting that some proteins may not stably associate with the IMD under this stress condition. Finally, in starved cells alkDADA incorporation appeared to decrease, and it labeled almost exclusively the septa, with some cells having multiple septa at various fluorescence intensities (Fig. 2D; Fig. S1). Taken together, these data suggest that the IMD and its associated enzymes are spatially rearranged and maintained under two different nongrowing conditions, stationary phase and starvation, perhaps to support nonpolar cell envelope biosynthetic activities, such as septal PG synthesis.

10.1128/mBio.01823-17.2 FIG S2  PBST starvation does not produce shorter cells. (A) Growth curve for cells after medium replacement with either PBST (starvation) or fresh Middlebrook 7H9 medium (control) (*n* = 3 cultures). (B) Cell length measurements (in micrometers) from the start (0 h) to the end (70 h) of PBST starvation, demonstrating stable cell lengths without substantial elongation or reduction (*n* = 174 cells). Download FIG S2, TIF file, 6.9 MB. Copyright © 2018 Hayashi et al. 2018 Hayashi et al. This content is distributed under the terms of the Creative Commons Attribution 4.0 International license.

10.1128/mBio.01823-17.3 FIG S3  PBST starvation leads to decreased IMD polar enrichment over time. Cap-to-sidewall ratios of fluorescence for HA-mCherry-GlfT2 (upper graph) and Ppm1-mNeonGreen-cMyc (lower graph) were measured at 6, 20, 48, and 70 h post-nutrient deprivation and demonstrated a progressive pattern of decreasing polar enrichment, which correlated with the prolonged starvation. The orange line represents the average cap-to-sidewall ratio of logarithmically grown cells for each fluorescent protein (*n* = 59 cells). Download FIG S3, TIF file, 9.8 MB. Copyright © 2018 Hayashi et al. 2018 Hayashi et al. This content is distributed under the terms of the Creative Commons Attribution 4.0 International license.

### Reduced PG biosynthesis triggers spatial rearrangement of the IMD.

**FIG 3:**
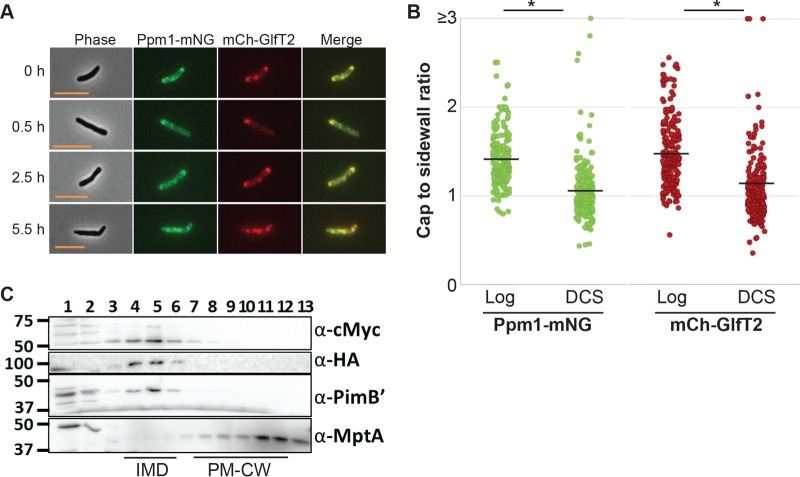
Inhibition of PG synthesis by DCS leads to reorganization of the IMD. (A) Fluorescence microscopy images of the dual IMD marker strain expressing HA-mCherry-GlfT2 (mCherry-GlfT2) and Ppm1-mNeonGreen-cMyc (Ppm1-mNG). DCS treatment led to the relocalization of the IMD from polar to sidewall enrichment. Scale bar, 5 µm. (B) The cap-to-sidewall ratio of two marker proteins in cells treated with DCS for 6 h compared with cells before treatment (log), quantitatively demonstrating reorganization from the polar cap to sidewall enrichment. The black lines indicate the averages of 218 cells. (C) Western blotting detection of IMD proteins (Ppm1-mNeonGreen-cMyc, 59 kDa; HA-mCherry-GlfT2, 100 kDa; PimB′, 42 kDa) and the PM-CW protein (MptA, 54 kDa) which were separated by sucrose density gradient sedimentation, illustrating enrichment in IMD fractions after 8 h of DCS treatment. *, *P* < 0.001.

As mentioned above, MurG is an IMD-associated glycosyltransferase involved in PG biosynthesis (13). Consistent with the association of this critical enzyme with the IMD, our results indicated that IMD enrichment at the cell poles correlates with polar PG biosynthesis. These observations implied that active PG synthesis at the poles could be a prerequisite for the polar enrichment of the IMD. However, it is also possible that IMD enrichment at the pole is triggered by different metabolic cues regardless of polar PG biosynthesis activity. To distinguish these possibilities, we tested if blocking PG biosynthesis triggered the delocalization of the IMD from the polar region. We inhibited PG biosynthesis chemically by using d-cycloserine (DCS), an inhibitor of d-alanine:d-alanine ligase and alanine racemase (21), which are involved in producing d-alanyl–d-alanine, a key substrate for the synthesis of the pentapeptide precursor. When the IMD reporter cell line was treated with 40 µg/ml DCS, the cells immediately ceased growth (Fig. S4A), and the IMD foci became delocalized from the polar region within 6 h of DCS treatment (Fig. 3A and B), similar to the responses found under stationary phase and starvation conditions. We examined the presence of the IMD in a sucrose gradient and found that it was maintained after DCS-induced inhibition of PG biosynthesis (Fig. 3C). These data indicated that perturbation of PG synthesis triggers the delocalization of the IMD from the pole.

10.1128/mBio.01823-17.4 FIG S4  Growth arrest by the inhibition of PG biosynthesis. (A) Growth curve of the dual IMD marker strain expressing HA-mCherry-GlfT2 and Ppm1-mNeonGreen-cMyc, treated with 40 µg/ml DCS to demonstrate growth arrest by an antibiotic targeting PG biosynthesis (*n* = 3 cultures). (B) Growth curve of the DAP auxotroph (mc^2^1620) upon replacing with the medium with (+) or without DAP (−), demonstrating the inhibitory effects of DAP removal (*n* = 3 cultures). Download FIG S4, TIF file, 7.3 MB. Copyright © 2018 Hayashi et al. 2018 Hayashi et al. This content is distributed under the terms of the Creative Commons Attribution 4.0 International license.

**FIG 4:**
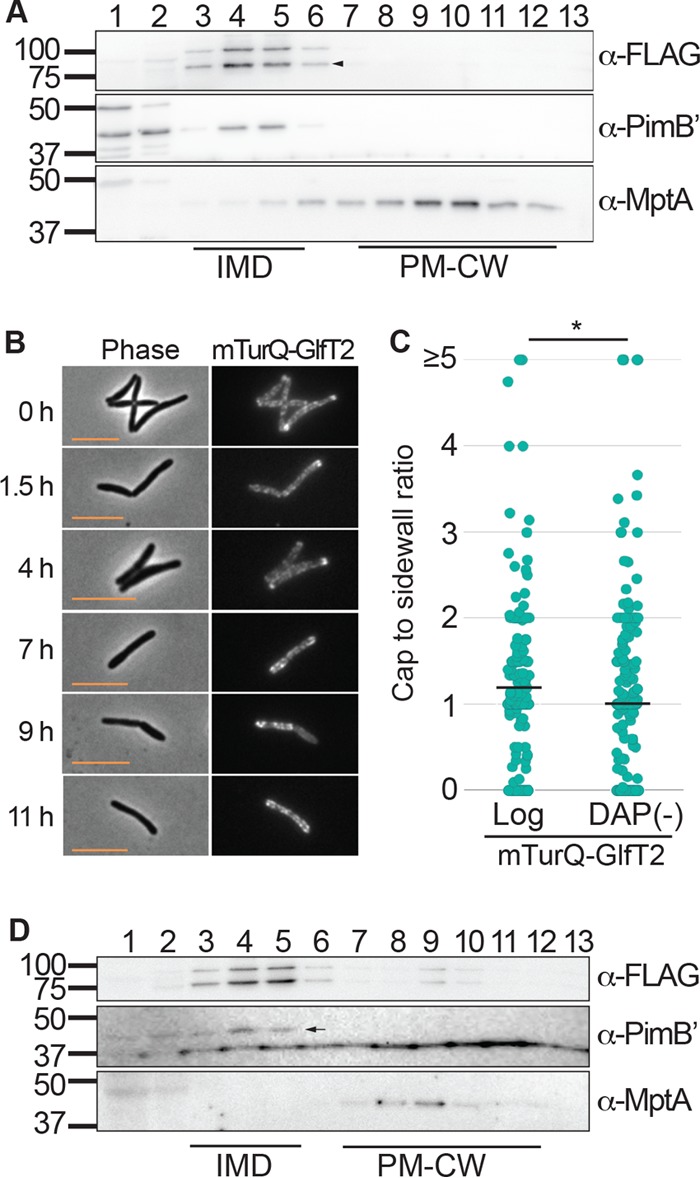
Genetic inhibition of PG synthesis leads to redistribution of the IMD. (A) Western blot detection of IMD proteins (mTurquoise-GlfT2-FLAG [mTurQ-GlfT2], 100 kDa; PimB′, 42 kDa), and PM-CW protein (MptA, 54 kDa) in the DAP auxotroph (strain mc^2^1620) expressing a fluorescent IMD marker (mTurQ-GlfT2-FLAG). The IMD fractions (4 to 6) show enrichment of both mTurQ-GlfT2-FLAG and PimB′ when grown with DAP-supplemented medium. Arrowhead, a degradation product which continues to associate with the IMD fraction. (B) Fluorescence microscopy images, demonstrating the relocalization of the IMD from polar to sidewall enrichment over 11 h of DAP deprivation. Scale bar, 5 µm. (C) Quantitative comparison of cells before treatment (log) and those after 10 h of DAP depletion [DAP(−)], demonstrating reorganization of mTruQ-GlfT2-FLAG from polar to sidewall enrichment. The black lines indicate the averages of 204 cells. (D) Western blotting detection of IMD proteins. Cells were starved for DAP for 10 h, and lysate was separated by sucrose density gradient fractionation. Arrow, PimB′. A band below PimB′ of nonspecific binding was occasionally seen under stress conditions. *, *P* < 0.001.

To confirm that the rearrangement of the IMD is not due to an off-target effect of DCS, we inhibited PG biosynthesis genetically by using a diaminopimelate (DAP) auxotroph. This mutant cannot synthesize DAP, an essential amino acid for formation of the PG pentapeptide and therefore requires an exogenous supply of DAP. We transformed this strain with a vector to express the previously established IMD marker (mTurquoise-GlfT2-FLAG) and tracked the subcellular localization of the IMD during logarithmic growth in the presence of an exogenous supply of DAP or under DAP depletion. As expected, the IMD marker was associated with the IMD fractions on the sucrose gradient (Fig. 4A, fractions 4 to 6) and enriched at the poles when the cells were actively elongating (Fig. 4B and C). However, when the medium was depleted of DAP, the cells ceased to grow at 8 h (Fig. S4B). Furthermore, at approximately the same time as the cells stopped growing, the IMD marker relocalized along the cell sidewall (Fig. 4B and C). Importantly, the IMD fractions remained isolatable by density gradient fractionation (Fig. 4D, fractions 4 to 6). These observations recapitulate the results obtained using DCS, suggesting that reduced PG biosynthesis is one critical factor that leads to delocalization of the IMD from the pole. Importantly, the IMD is spatially rearranged but maintained as a biochemically isolatable membrane domain.

While these data suggest that ongoing PG synthesis is a prerequisite for polar IMD localization, PG biosynthesis may not be the sole factor for the spatial rearrangement. The spatial change of the IMD in response to growth-phase transition and nutrient deprivation suggests a more global metabolic response. Indeed, when we added isoniazid (INH), an inhibitor of fatty acid biosynthesis, we observed a similar delocalization of the IMD within 5.5 h at two different concentrations of the drug (Fig. S5). These observations, generated using different chemical and genetic inhibition methods, suggest that the subcellular localization of the IMD is likely governed by the general metabolic status of the cell.

10.1128/mBio.01823-17.5 FIG S5  INH treatment leads to alterations in IMD localization. (A) INH at two different concentrations (50 or 100 µg/ml) caused slight delays in growth compared to untreated cells. (B to D) Fluorescent images demonstrate the spatial changes in IMD localization in cells treated with 0, 50, or 100 µg/ml INH, respectively. Scale bar, 5 µm. Download FIG S5, TIF file, 9.6 MB. Copyright © 2018 Hayashi et al. 2018 Hayashi et al. This content is distributed under the terms of the Creative Commons Attribution 4.0 International license.

### Polar enrichment of the IMD is restored upon reinitiation of growth.

**FIG 5:**
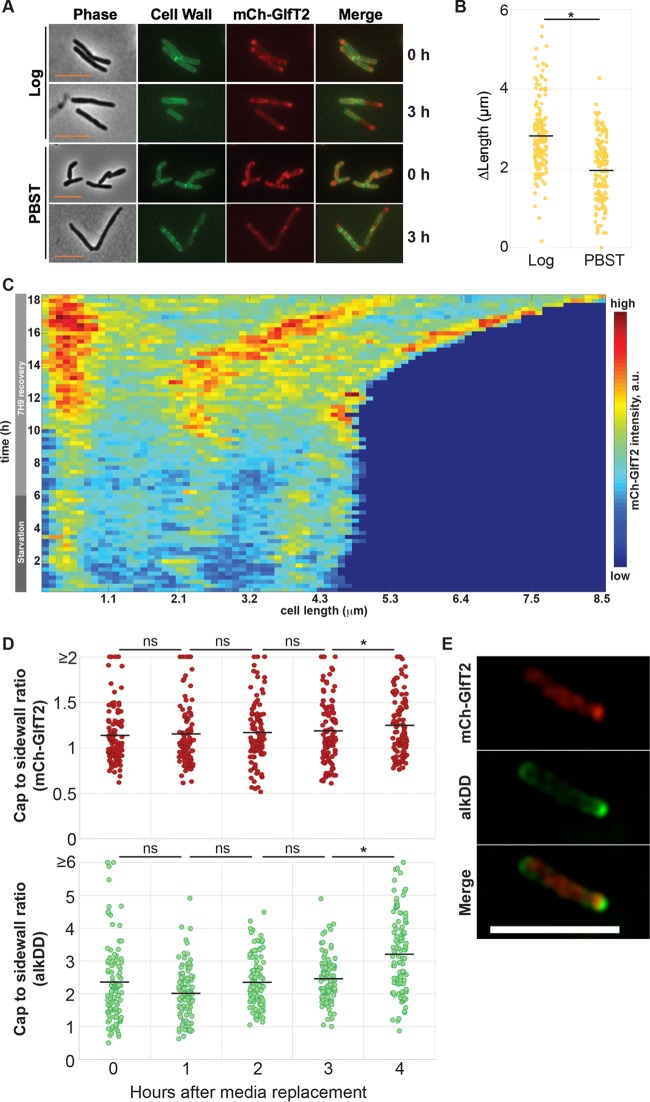
Polar enrichment of the IMD is restored after starvation and correlates with the initiation of PG synthesis and cell elongation. (A) Fluorescence live imaging of logarithmically growing (log) and starved cells (PBST, 30 h) immediately after cell surface staining with amine-reactive Alexa Fluor 488 fluorescent dye (0 h) or after being grown in fresh medium for 3 h. The single IMD marker strain expressing HA-mCherry-GlfT2 alone was used. Polar regions unstained by the amine-reactive dye indicate the areas of cell envelope growth during the 3-h growth period. Scale bar, 5 µm. (B) Cell envelope elongation after a 3-h recovery period, demonstrating that greater elongation is observed in actively growing cells (log) than in the cells recovering from PBST starvation. The black lines indicate the averages of 204 cells. (C) Kymograph from time-lapse imaging of the dual IMD marker strain expressing HA-mCherry-GlfT2 and Ppm1-mNeonGreen-cMyc and starved in PBST for 6 h and recovered in fresh Middlebrook 7H9 medium, showing recovery of the polar IMD after ~4 h of medium replacement and subsequent cell growth. A representative cell is shown, and others are shown in Fig. S6. The darkest blue (lower right) demarks areas of the graph beyond the length of the cell at that time point. (D) Polar enrichment (cap/sidewall ratio) of the IMD (mCherry-GlfT2) and PG synthesis (alkDADA) increases at the 4-h recovery time point after 6 h of starvation in PBST. The single IMD marker strain expressing HA-mCherry-GlfT2 was used. Representative images are also shown in Fig. S6. The black lines indicate the averages of 201 cells. (E) SIM images of the single IMD marker strain recovering from 6 h of PBST starvation, demonstrating the polar PG synthesis (alkDADA) and the IMD enrichment (mCh-GlfT2) slightly subpolar and adjacent to the PG synthesis after 4 h of recovery. *, *P* < 0.001; ns, not significant. Scale bar, 5 µm.

We have so far demonstrated that the IMD reorganizes in cells that are not growing. We next asked if the IMD restores the polar localization when cells are returned to a nutrient-replete condition. To monitor cell envelope elongation during recovery from starvation, we used an amine-reactive dye to stain the existing cell walls of starved cells (30 h in PBST) and placed them in fresh Middlebrook 7H9 medium. We determined the resumed envelope elongation by using fluorescence microscopy (Fig. 5A and B). As a control, logarithmically growing cells continued to elongate, with an average extension of 2.8 ± 0.88 µm (mean ± standard deviation) after 3 h of growth in fresh medium. In contrast, the starved cells added only 1.9 ± 0.81 µm to their total cell length during the same 3-h period under the nutrient-replete condition (Fig. 5B). The IMD in starved cells (in PBST) was less enriched in the polar region prior to recovery and became more enriched at 3 h post-nutrient repletion (Fig. 5A), suggesting that cell envelope elongation is slower or delayed in the initial recovery phase after starvation, when polar enrichment of the IMD is not fully restored.

To provide further insights, we used time-lapse microscopy and monitored the IMD in cells starved in PBST for 6 h and then allowed to recover in fresh Middlebrook 7H9 medium (Fig. 5C). In most cells (25 of 29), we observed a pattern of mild IMD polar enrichment and minimal cell growth during PBS starvation (Fig. 5C; Fig. S6). In these cells, we observed polar enrichment within ~4 h of recovery, followed by cell growth within ~6 h of recovery (Fig. 5C; Fig. S6). To determine the location of PG synthesis, we probed recovering cells with alkDADA (Fig. S7). Under this growth condition, the IMD started to become more enriched in the polar region at 4 h post-nutrient repletion (Fig. 5D; Fig. S7). Importantly, this is the same time point when PG metabolic labeling becomes more prominently enriched in the polar region. Structural illumination microscopy (SIM) revealed that the enrichment of the IMD and the enriched synthesis of PG did not colocalize, but the IMD was distinctively subpolar to the PG synthesis (Fig. 5E). Taken together, enrichment of the polar IMD is spatiotemporally correlated with the initiation of polar PG synthesis and cell elongation.

10.1128/mBio.01823-17.6 FIG S6  IMD localization and growth during starvation and recovery, visualized using time-lapse microscopy. The dual IMD marker strain expressing HA-mCherry-GlfT2 and Ppm1-mNeonGreen-cMyc was starved in PBST for 6 h and then allowed to recover in Middlebrook 7H9 by using a microfluidic system. Images were recorded every 15 min, and 29 cells were analyzed. (A) Linear growth rate, averaged over two frames (30 min total) through the time-lapse imaging. (B to E) Kymograph of four cells. Panels B to D show recovery of the polar IMD ~4 h after medium replacement and subsequent cell growth similar to the cell shown in Fig. 5C, while panel E shows an example of the rare cells (4 of 29) where IMD polarity and growth in recovery were not correlated. The darkest blue (lower right) demarks areas of the graph beyond the length of the cell at that time point. Download FIG S6, TIF file, 15.5 MB. Copyright © 2018 Hayashi et al. 2018 Hayashi et al. This content is distributed under the terms of the Creative Commons Attribution 4.0 International license.

10.1128/mBio.01823-17.7 FIG S7  Polar IMD enrichment correlates with enriched polar PG synthesis. Fluorescence microscopy images of cells starved in PBST for 6 h and recovered in Middlebrook 7H9 medium (0 to 4 h) are shown and demonstrate the restoration of polar IMD and PG synthesis over the recovery period. Download FIG S7, TIF file, 12.1 MB. Copyright © 2018 Hayashi et al. 2018 Hayashi et al. This content is distributed under the terms of the Creative Commons Attribution 4.0 International license.

## DISCUSSION

The IMD is a growth pole enriched membrane domain in mycobacteria. Previously, we demonstrated that the IMD houses an array of biosynthetic activities that are essential for cell envelope biogenesis (14). The metabolically active nature of the IMD led us to question how it reacts to environmental conditions that inhibit bacterial growth. Therefore, in this study we focused on short-term stress responses and aimed to determine the nature of the IMD under nonelongating conditions.

We tested two models, in the stationary growth phase and under PBST starvation, which entail distinct cellular responses. In stationary phase, mycobacteria no longer elongate but they continue to separate into smaller cells (21). Therefore, cell envelope components are needed for septum synthesis. We confirmed the generation of shorter cells in stationary phase and further demonstrated the spatial delocalization of the IMD from the pole. Importantly, the IMD was purified from the stationary-phase cells by density gradient fractionation, suggesting that it was maintained as a membrane domain. We also tested starvation as another model of stress exposure, using a recently published mild starvation model (18) in which cells are starved in PBST. In this model, mycobacteria initially respond by generating septa without cell separation, creating relatively long cells with multiple septa. Similar to the spatial reorganization in stationary-phase cells, we found that the IMD is reorganized to the sidewall of the cell and can still be biochemically isolated by gradient fractionation. Thus, in both of these stress models, the IMD is maintained as a membrane domain but is spatially delocalized from the polar region.

While the IMD is maintained as a membrane domain, not all proteins continue to localize to the IMD. For example, a fraction of PimB′ was present in the cytosol and the conventional plasma membrane in stationary growth phase. More prominent relocalization of Ppm1-mNeonGreen-cMyc to the conventional plasma membrane was also detected in starved cells. These data imply that each IMD-associated protein responds differently to the stress conditions and can change its subcellular localization even when the IMD is still present for binding. PimB′ is a peripheral membrane protein, while Ppm1 binds to the membrane by forming a heterodimer with a membrane protein, Ppm2 (22, 23). How certain proteins are released from the IMD during specific growth-arresting conditions may be dependent on how proteins associate with the membrane. Our data are generally consistent with the idea that the IMD is physically maintained under nongrowing conditions and is relocated spatially in response to stress conditions. However, our current data do not exclude an alternative possibility, that the polar IMD enriched in the growing cells is turned over by degradation and regenerated in nonpolar locations under stress conditions. Distinguishing these two alternative possibilities is another important point to be addressed in the future.

The dynamic spatial rearrangement of the IMD suggests that septal synthesis could be part of the reason why the membrane domain rearranges in these stress models. To address the link between septal synthesis and the IMD rearrangement more directly, we metabolically labeled PG and showed in both stationary-phase and starved cells that PG synthesis actively takes place at the septa. While our present model ties PG synthesis to the spatial alterations in the IMD, a similar reorganization of the IMD was also evident upon treatment with INH. Therefore, our data are consistent with the idea that growth inhibition in general is a trigger of IMD reorganization. We speculate presence of a master regulator that triggers the IMD rearrangement, but its identity and the downstream molecular events involved remain to be determined.

Stationary-phase cells undergo cell division without elongation. We found some polar alkDADA incorporation in stationary-phase cells, but we considered this polar labeling to be residual incorporation of the probes into nascent poles created by the cell separation. In contrast, starvation in PBST did not induce significant levels of production of shorter cells, and alkDADA incorporation was found predominantly in septa. These observations are consistent with an earlier observation of multiseptated cells after PBST starvation (18). Importantly, the IMD was often enriched near the site of PG synthesis in both models, highlighting the correlation between septal synthesis and IMD rearrangement under stress conditions.

Previously, we showed a correlation between polar cell envelope elongation and the localization of the IMD at the polar region. Furthermore, the IMD also accumulated at drug-induced ectopic growth poles (14). These previous observations, together with the current results of near-septal IMD formation under stress conditions, suggest a model in which there is a tight spatial link between the IMD and the site of incorporation of the nascent PG precursor to the existing cell wall. Interestingly, MurG, a glycosyltransferase involved in lipid II synthesis, is an IMD-associated enzyme (13). Therefore, we speculate that the IMD plays a key role in facilitating the localized production of PG precursors at sites where they are needed.

In the current study, we further showed that chemical and genetic inhibition of PG synthesis are sufficient to delocalize the IMD from the polar region. Such delocalization took place in a nutrient-rich medium, implying that suppression of PG synthesis could be a dominant signal that can trigger the IMD delocalization despite the availability of nutrients. A serine/threonine protein kinase, such as PknB, which has an extracytoplasmic sensor domain with potential roles in detecting PG precursors (24), might mediate the initial signal transduction that leads to the observed rearrangement of the IMD. Importantly, functions of the IMD are diverse, and how metabolic changes other than PG biosynthesis can impact the subcellular localization of the IMD remain to be examined.

Our previous observation of IMD enrichment at the ectopic growth poles implied the existence of mechanisms to reconstruct the polar IMD after stress (14). Supporting this observation, in this study we demonstrated that polar enrichment is recoverable after a different stress, and the relative level of polar enrichment appears to be correlated with PG synthesis and growth rate. We determined that PBST-starved cells elongate at about two-thirds the rate of those with IMD enrichments on average in the initial 3-h recovery period (Fig. 5A and B). These data suggest that the difference in the rate of cell envelope elongation is linked to the timing of the spatial enrichment of the IMD. In summary, our current study substantiates the concept that polar IMD enrichment may be a strategy for mycobacteria to concentrate metabolic machinery so that they can achieve rapid and efficient growth.

## MATERIALS AND METHODS

### Cell strains, cultures, and growth curve generation.

Markerless knock-in *M. smegmatis* strains expressing both HA-mCherry-GlfT2 and Ppm1-mNeonGreen-cMyc (SUMA187) or HA-mCherry-GlfT2 alone (SUMA86) were previously established (13). These strains were grown in Middlebrook 7H9 medium supplemented with 0.05% Tween 80, 11 mM glucose, and 14.5 mM NaCl at 30°C with shaking. Growth was monitored via the OD_600_. For starvation experiments, *M. smegmatis* cultures were grown to logarithmic phase, spun down to remove the medium, and resuspended in PBS supplemented with 0.05% Tween 80. The DAP auxotroph (mc^2^1620) was transfected with the pMUM43 vector to express mTurquoise-GlfT2-FLAG (13). The DAP auxotroph was grown in Middlebrook 7H9 supplemented with Middlebrook ADC supplement, amino acids (l-lysine, l-methionine, and l-threonine at 40 mg/ml and dl-homoserine at 80 µg/ml), and DAP (200 µg/ml) as previously described (25).

### Click-iT chemistry and alkDADA incorporation.

*M. smegmatis* cells were grown in medium supplemented with 2 mM alkDADA for 15 min at 30°C, fixed in 2% formaldehyde, and probed with the Alexa Fluor 488 picolyl azide (Thermo Fisher) for 30 to 60 min at room temperature in the dark.

### Inhibition of PG biosynthesis.

For chemical inhibition, cells were treated with 40 µg/ml DCS. For genetic inhibition, the DAP auxotroph cells were spun down and resuspended in medium lacking DAP.

### Cell wall staining.

*M. smegmatis* cells were treated with 5 mg/ml Alexa Fluor 488 NHS ester (succinimidyl ester; Thermo Fisher) for 1 min at room temperature in the dark, washed with PBST, returned to fresh Middlebrook 7H9, and incubated further at 30°C with shaking. After 3 h of growth, cells were fixed in 2% formaldehyde and washed with PBST.

### Fluorescence microscopy and image analysis.

To visualize fluorescence, a 2 μl aliquot of live or fixed *M. smegmatis* cells was spotted onto a 1% agar pad with water and covered with a glass coverslip. All static live cell images were taken under identical settings (100× objective and 175-ms exposure for phase-contrast microscopy, or 3-s exposure for fluorescence), using a Nikon Eclipse E600 microscope equipped with an ORCA-ER cooled charge-coupled-device camera (Hamamatsu) and Openlab software 5.5.2 (Improvision). Mean fluorescence intensity of alkDADA incorporation was calculated using Fiji (26), and the average fluorescence is indicated in the figures by a black bar. Polar enrichment was measured using a custom MatLab program that calculated mean fluorescence of the pixels at the pole (designated 10 px from the polar end), compared to the average sidewall pixel intensity (intensity of total cell minus the identified polar ends). Cell length was measured from phase-contrast images using Fiji. Statistical significance was determined via a *t* test. Time-lapse microscopy was performed as previously described (13, 27). Briefly, cells were loaded into a custom constant flow microfluidic device (11, 27) and observed by a DeltaVision PersonalDV wide-field fluorescence microscope in a controlled environmental chamber warmed to 37°C. SIM images were acquired via a Nikon Eclipse Ti N-SIM E microscope equipped with a Flash4.0 camera and a numerical aperture of 1.49. Images were taken at 100-ms exposures and reconstructed using NIS-Elements.

### Density gradient fractionation.

For density gradient fractionation, cells were pelleted, washed in 50 mM HEPES (pH 7.4) buffer, and resuspended in lysis buffer (25 mM HEPES [pH 7.4], 20% sucrose, 2 mM EGTA, and a protease inhibitor cocktail) at 1 g (wet weight) of pellet in 4 ml of lysis buffer. The cell suspension was then subjected to nitrogen cavitation at ~2,250 lb/in^2^ for three times for 30 min each. The lysate was spun at 3,220 × *g* for 10 min at 4°C twice before it was loaded on a 20-to-50% sucrose gradient. Gradients were spun at 218,000 × *g* for 6 h at 4°C, fractionated into 1-ml fractions, and used for further biochemical analyses.

### SDS-PAGE and Western blotting.

Protein samples were mixed with a reducing sample loading buffer, denatured on ice, and run in a 12% SDS-PAGE gel. Proteins were transferred to a polyvinylidene difluoride membrane (Bio-Rad) and blocked in 5% milk in PBST20 (PBS with 0.05% Tween 20). Membranes were incubated with primary antibodies (anti-HA [Sigma], anti-PimB′ [28], and anti-MptA [28]) at 1:2,000 dilutions, washed in PBST20, and incubated with horseradish peroxidase-conjugated secondary antibodies (GE Healthcare) at 1:2,000 dilutions. Blots were washed again in PBST20 and developed for chemiluminescence. Images were recorded using an ImageQuant LAS 4000mini (GE Healthcare). For Western blotting of sucrose gradients, an equal volume of each fraction was loaded into the gel. All experiments were repeated at least twice.
