# Supplementary figures and images for: Stress-Induced Reorganization of the Mycobacterial Membrane Domain
Source: mBio. 2018 Jan 23;9(1):e01823-17. doi: 10.1128/mBio.01823-17 (PMC5784251; doi:10.1128/mBio.01823-17)

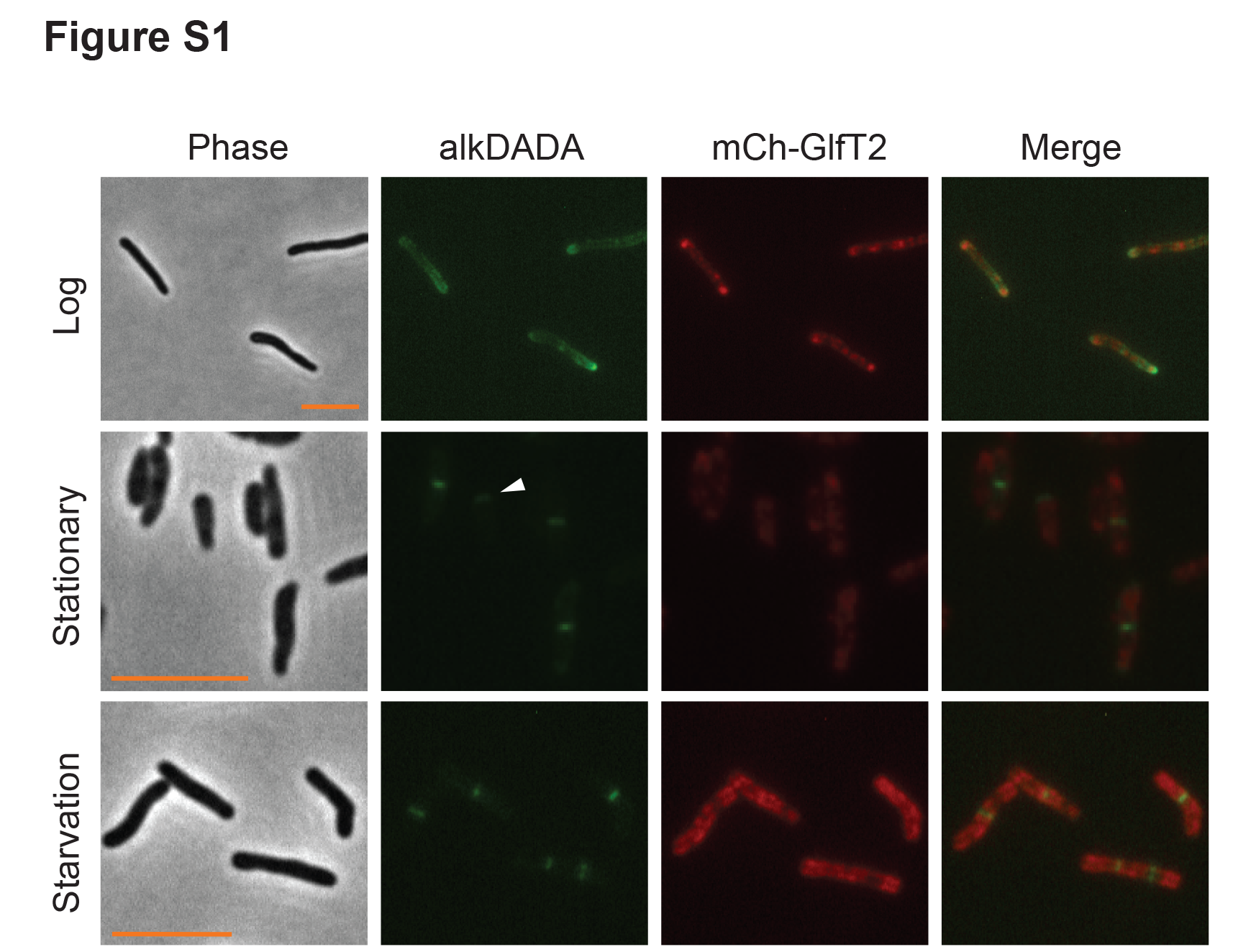

Supplement: FIG S1 [file mbo001183676sf1.tif]

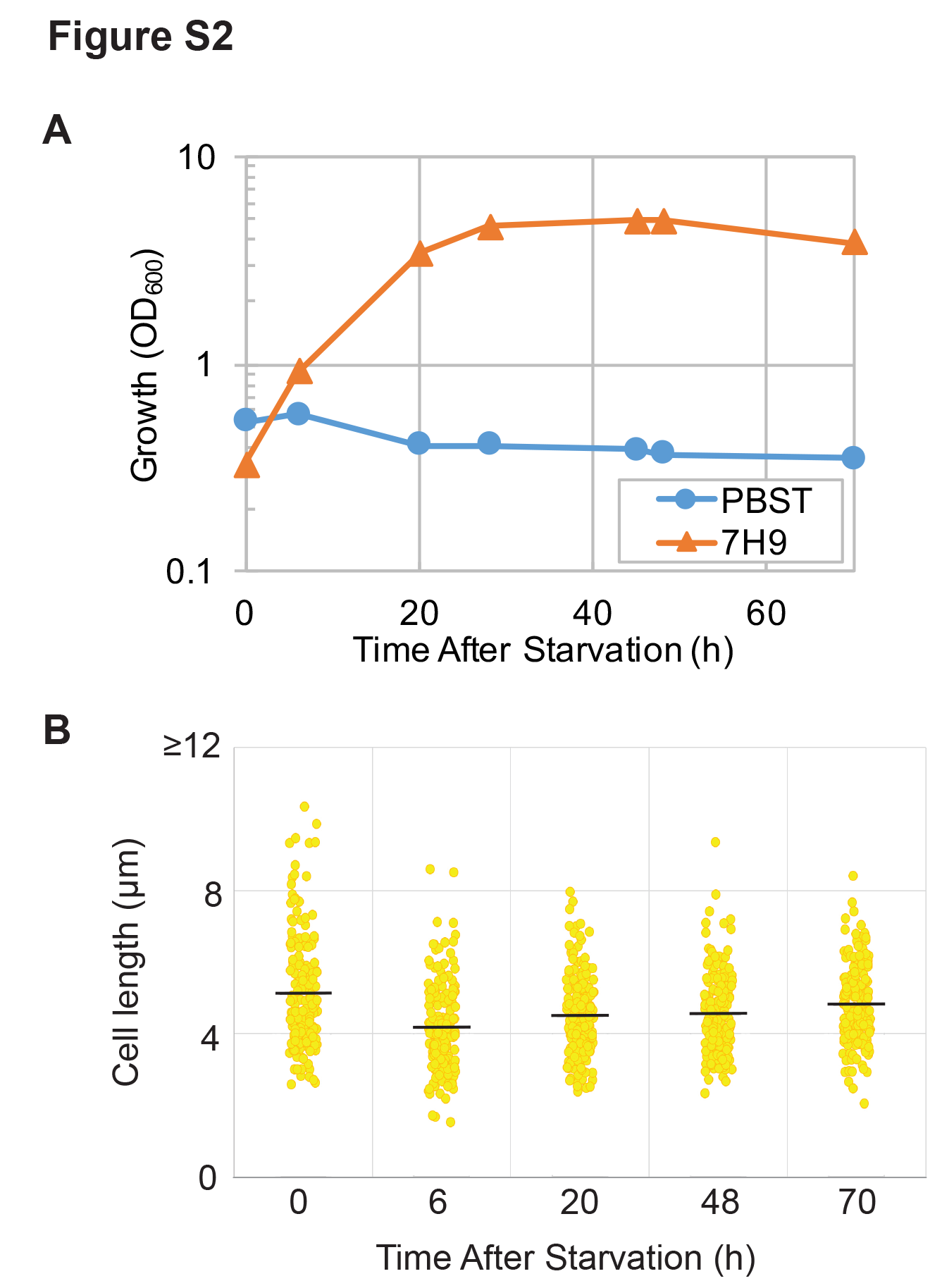

Supplement: FIG S2 [file mbo001183676sf2.tif]

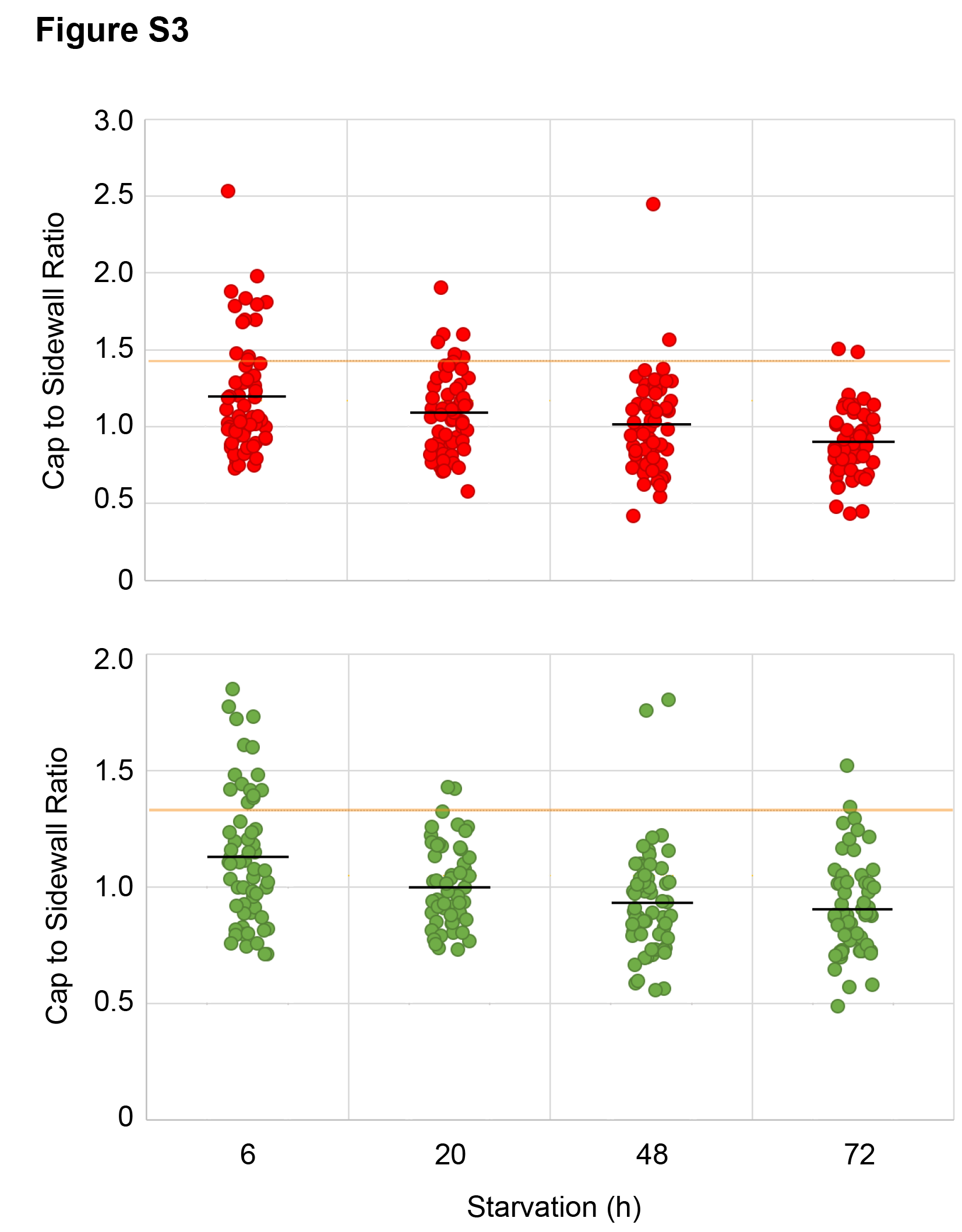

Supplement: FIG S3 [file mbo001183676sf3.tif]

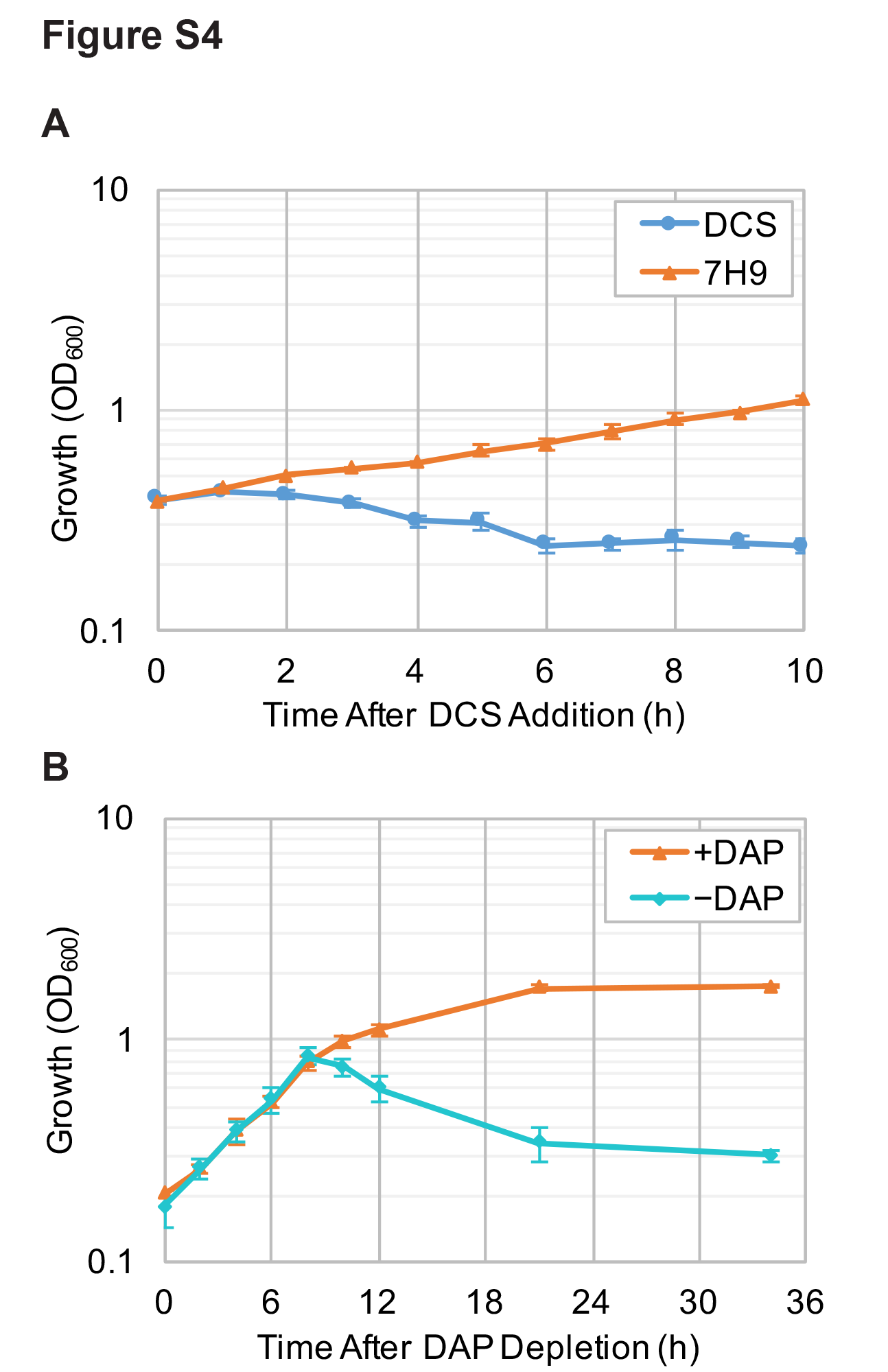

Supplement: FIG S4 [file mbo001183676sf4.tif]

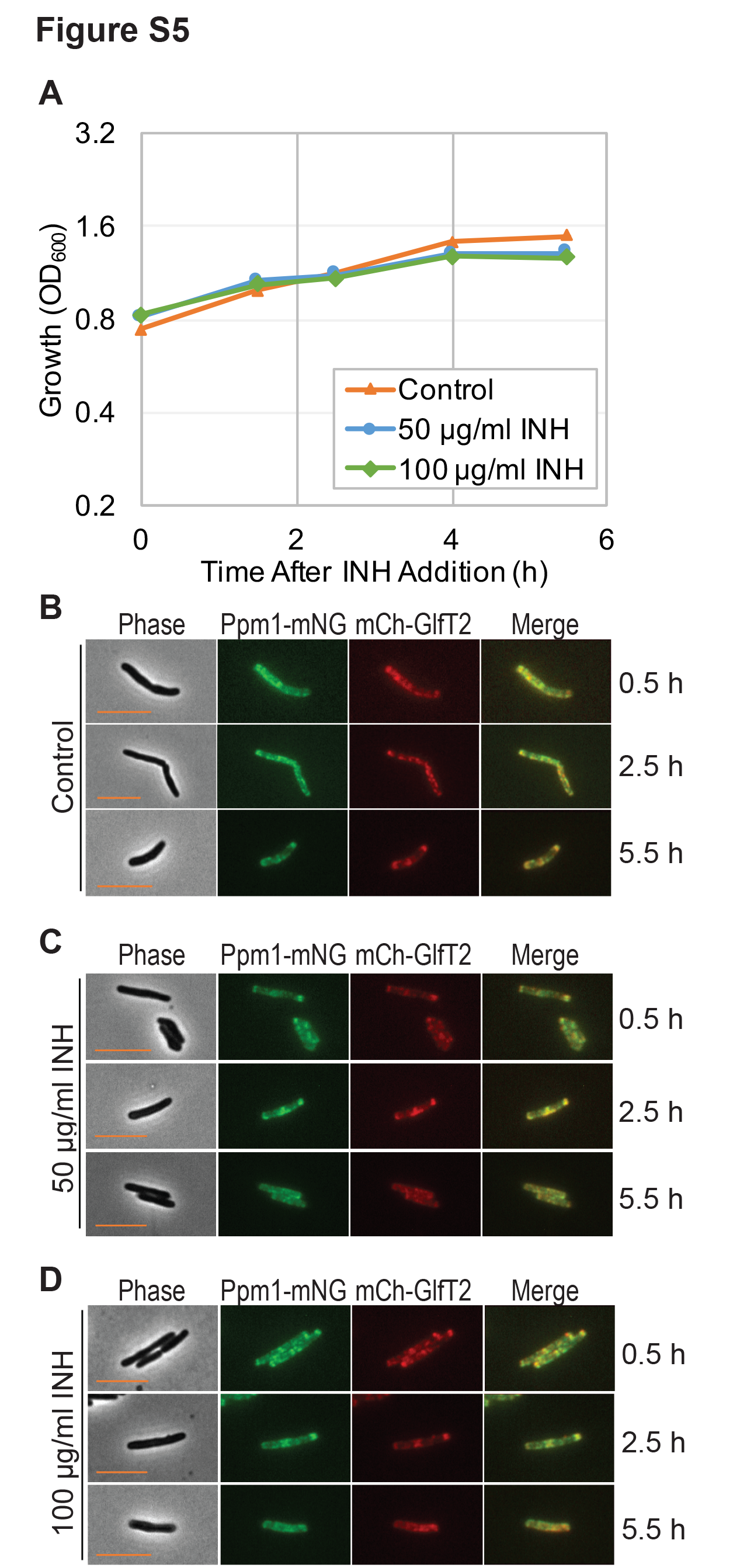

Supplement: FIG S5 [file mbo001183676sf5.tif]

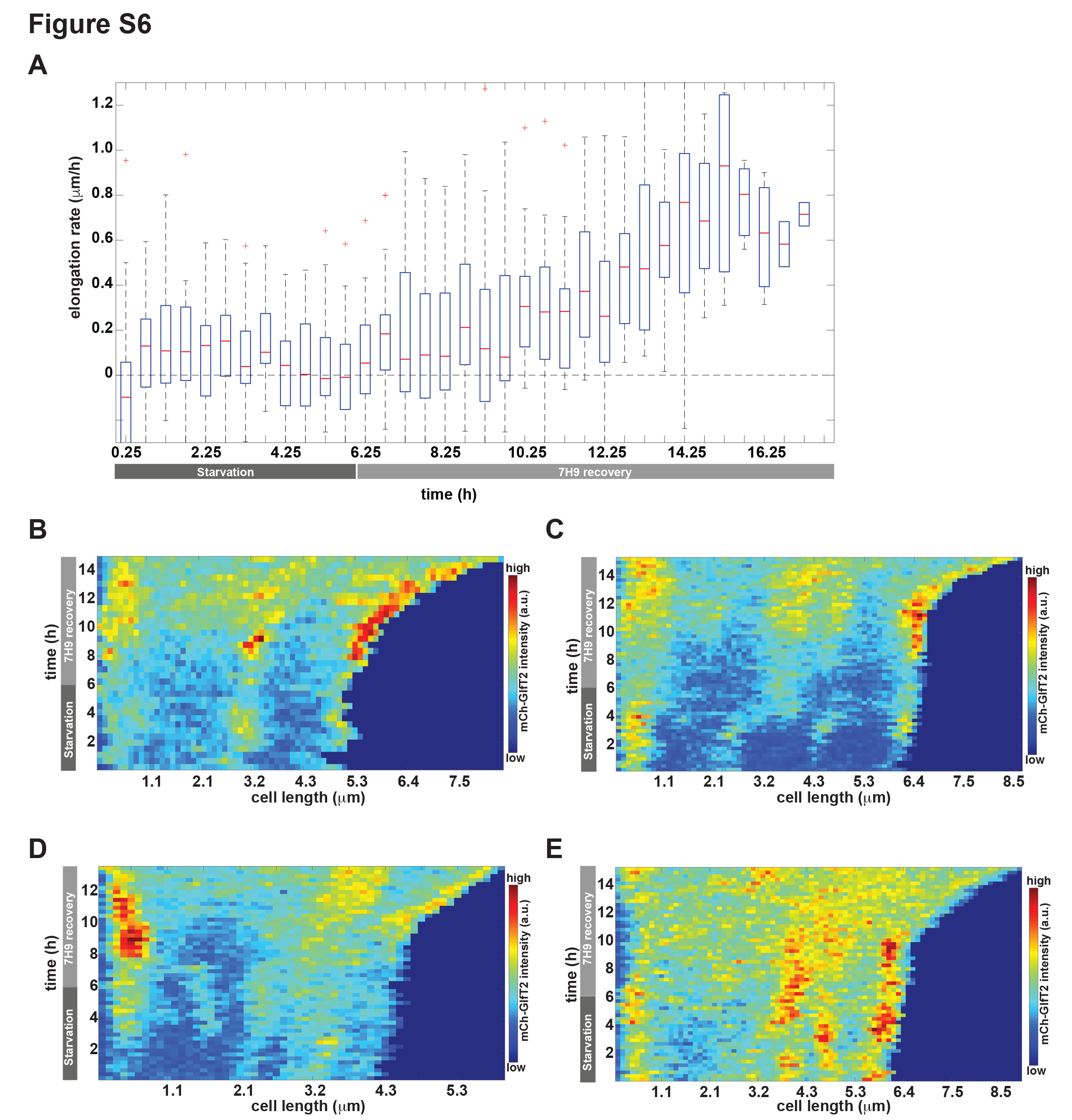

Supplement: FIG S6 [file mbo001183676sf6.tif]

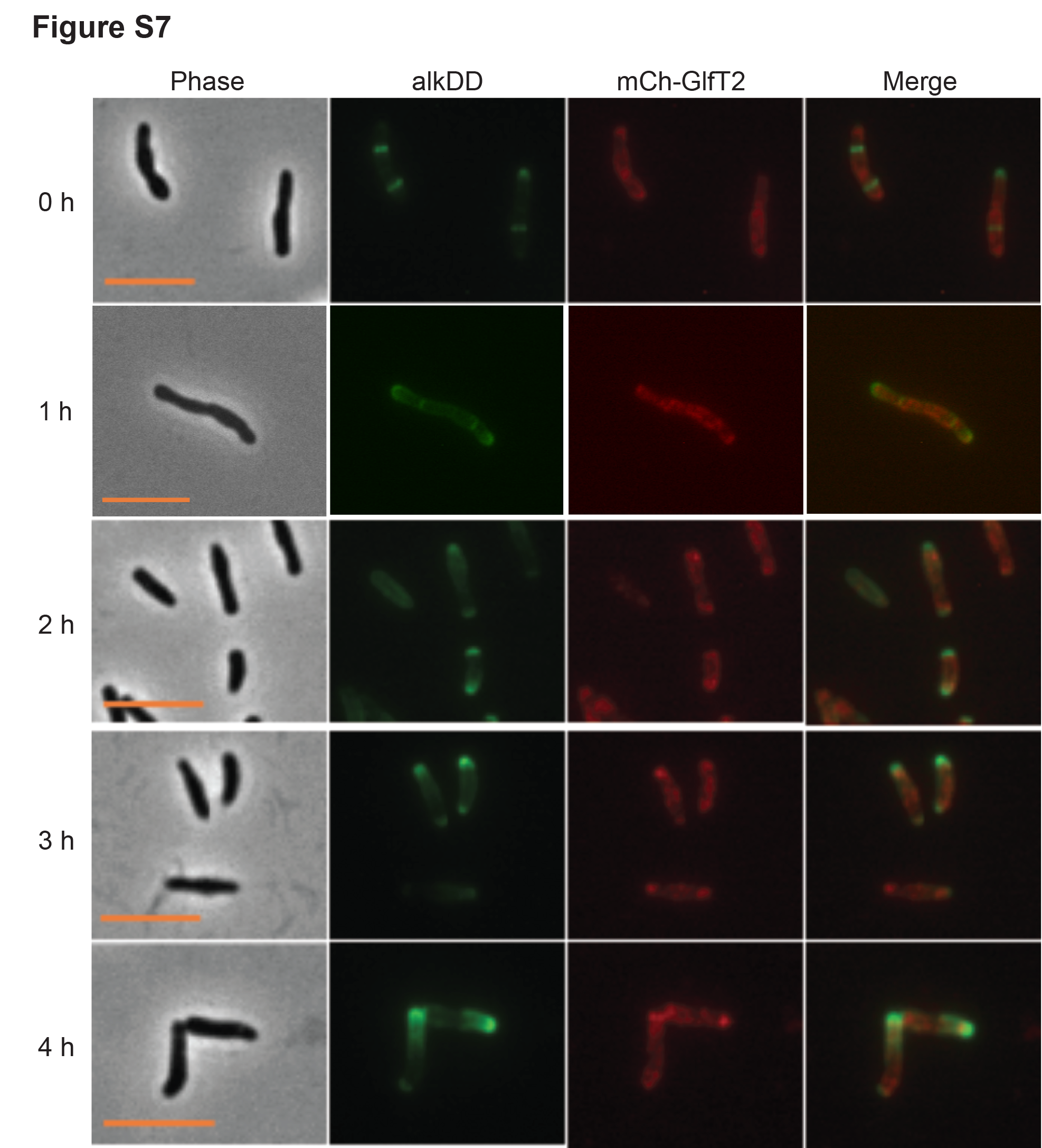

Supplement: FIG S7 [file mbo001183676sf7.tif]
